# Interaction of *Bordetella bronchiseptica* and Its Lipopolysaccharide with *In Vitro* Culture of Respiratory Nasal Epithelium

**DOI:** 10.1155/2013/347086

**Published:** 2013-03-11

**Authors:** Carolina Gallego, Andrew M. Middleton, Nhora Martínez, Stefany Romero, Carlos Iregui

**Affiliations:** ^1^Department of Veterinary Pathology, University of Applied and Environmental Sciences, Bogotá, Colombia; ^2^GlaxoSmithKline Consumer Healthcare, St George's Avenue, Weybridge, Surrey KT13 0DE, UK; ^3^Laboratory of Veterinary Pathology, Faculty of Veterinary Medicine, National University of Colombia, Bogotá, Colombia

## Abstract

The nasal septa of fetal rabbits at 26 days of gestation were harvested by cesarean section of the does while under anesthesia and then exposed to *Bordetella bronchiseptica* or its lipopolysaccharide (LPS) for periods of 2 and 4 hours. A total of 240 explants were used. The tissues were examined using the Hematoxylin & Eosin technique. Then, semithin sections (0.5 *μ*m) were stained with toluidine blue and examined with indirect immunoperoxidase (IPI) and lectin histochemistry. The most frequent and statistically significant findings were as follows: (1) cell death and increased goblet cell activity when exposed to bacteria and (2) cell death, cytoplasmic vacuolation and infiltration of polymorphonuclear leukocytes when exposed to LPS. The lesions induced by the bacterium were more severe than with LPS alone, except for the cytoplasmic vacuolation in epithelial cells. IPI stained the ciliated border of the epithelium with the bacterium more intensely, while LPS lectin histochemistry preferentially labeled the cytoplasm of goblet cell. These data indicate that *B. bronchiseptica* and its LPS may have an affinity for specific glycoproteins that would act as adhesion receptors in both locations.

## 1. Introduction

*Bordetella bronchiseptica* is a Gram-negative bacterium capable of colonizing the respiratory tract of a large range of mammalian hosts, including mice, rats, guinea pigs, rabbits, cats, dogs, pigs, sheep, horses, and bears [1]. *B. bronchiseptica* is responsible for a wide spectrum of overt respiratory diseases such as kennel cough in dogs, atrophic rhinitis in pigs, and snuffles and pneumonia in rabbits [2–4]. *B. bronchiseptica *can also lead to permanent asymptomatic colonization of the respiratory tract [1, 5].

In dogs, *B. bronchiseptica* causes tracheobronchitis with two patterns of histological lesions. One pattern consists of focal areas of epithelial degeneration, necrosis, and cellular disorganization with vacuolation and pyknosis represented by congestion in the lamina propria, infiltrated with macrophages and lymphocytes. The second pattern consists of mucopurulent exudates that accumulate in the lumen of the airway with edema in the lamina propria, marked infiltration of polymorphonuclear leukocytes and clumps of bacteria located between the cilia of the tracheobronchial epithelium [10].

In piglets, *B. bronchiseptica *can cause upper respiratory illness, leading to nonprogressive atrophic rhinitis; histologically, other common lesions include hyperplasia of the epithelium with metaplasia and deciliated cells. In rabbits, *B. bronchiseptica* causes a suppurative bronchopneumonia with interstitial pneumonia; histologically, peribronchial lymphocytic cuffing has been described in [1, 11]. In species other than piglets [12–14], detailed descriptions of the initial changes in the respiratory epithelium of the nasal cavity have not been reported.

Numerous *B. bronchiseptica* virulence factors have been implicated as being responsible for damaging the host cells. These factors include toxins such as adenylate cyclase toxin, dermonecrotic toxin, the type III secretion system, and adhesins such as filamentous hemagglutinin, pertactin, and fimbriae [15, 16]. Most of the effects of these factors have been described from *in vitro* studies working with isolated cell cultures exposed to the respective virulence factor.

Despite the importance of *B. bronchiseptica *infection in rabbits [1, 17], as well as of the lipopolysaccharide (LPS) of *B. bronchiseptica *as a virulence factor, no reports on the effects of the whole microorganism or its LPS using a nasal septum culture have been documented in this species. Recent work from our group with another respiratory pathogen of rabbits, *Pasteurella multocida*, showed damage caused by the LPS of this pathogen to the respiratory epithelium of rabbit fetuses using the same model presented in this study [18]. In addition, the author found that the LPS of *P. multocida *significantly increased the number of bacteria adhering to the epithelium when this molecule was applied 30 minutes before or simultaneously with exposure to the microorganism. A similar effect has been described for the LPS of other Gram negative bacteria, such as *Salmonella enterica *[19] and *Helicobacter pylori *[20].

The goal of this work was to detail the changes caused by *B. bronchiseptica* in the respiratory epithelium of the nasal septum of rabbits during the first hours of infection. Additionally, we sought to examine whether the LPS of this pathogen by itself could cause lesions that would complicate the damages caused by the bacterium. To test this, a novel experimental approach more similar to natural conditions was used, namely, a tissue culture from the nasal septum of fetal rabbits.

## 2. Materials and Methods

### 2.1. Bacteria

*B. bronchiseptica *0301 was isolated from rabbits having clinical symptoms of rhinitis or pneumonia; the animals originated from commercial farms located on the flat plain near Bogotá, Colombia (2,600 m.a.s.l.). Samples were collected from nares or trachea, cultured on brain heart infusion agar (BHI), and were stored in 10% glycerol at −70°C until use.

### 2.2. LPS Production

#### 2.2.1. Bacterial Biomass Production

Large-scale virulent *B. bronchiseptica *cultures were collected in BHI agar and its biomass was harvested. The bacterium was suspended in distilled water (DW) with 0.1% thimerosal at 4°C for inactivation and conservation. The cells were centrifuged at 2,500 g for 50 min in sterile DW.

#### 2.2.2. LPS Extraction

Westphal and Jann's [24] phenol-hot water method was used. Inactivated bacteria were suspended in DW, treated with 90% phenol v/v for 30 min at 68°C, and stored at 4°C for 24 h. When phase separation (aqueous, phenolic interphase, and precipitate) was evident, the samples were centrifuged at 3,000 g for 30 min at 4°C [25]. The aqueous phase was separated and treated with 1 : 10 volume of 95% ethanol that was then stored for 18 h at −20°C and then centrifuged at 2,500 g for 15 min to obtain crude LPS. LPS precipitate was suspended in physiological saline solution (PSS), centrifuged at 2,000 g for 30 min, and then dialyzed against sterile DW. The LPS was quantified by the Lee and Tsai's colorimetric method [26]. LPS (25 *μ*g in 100 *μ*L PSS) was intraperitoneally inoculated into five mice to evaluate its biological activity. An additional five mice were injected with sterile PSS. The first five mice were euthanized when clinical signs appeared, and their tissues were processed by routine histopathological technique.

### 2.3. Hyperimmune Antisera Production

One mature sheep was used for hyperimmune antiserum production against *B. bronchiseptica *0301. Next, 35 days after the first inoculation of killed *B. bronchiseptica *0301 with complete Freund adjuvant was administered, one repetition with incomplete Freund adjuvant and one repetition with only the bacterium, the animal was bled. The antiserum was immunoadsorbed with fetal rabbit turbinate macerates to eliminate cross-reaction with rabbit tissues, and they were washed with PSS and diluted 1 : 25 in sterile buffer (Tris saline, pH 7.6). One mL of antiserum was admixed with 1 mL macerate, washed with PSS, and centrifuged at 133 g for 1 h at room temperature. The supernatant contained the immunoadsorbed antiserum and was kept at –20°C until use. The antiserum titer was calculated by indirect immunodot at 1 : 25–1 : 400 dilutions.

### 2.4. Fetal Rabbit Nasal Septum Culture

Twenty-six day pregnant does (*n* = 10, 240 explants) were subjected to cesarean section under aseptic conditions; they had been previously anesthetized with 5 mg/Kg xylazine, 35 mg/Kg ketamine. Fetuses were immediately euthanized by medullar sectioning. Three sequential 2-mm-thick transversal sections were obtained from the nasal cavity. The sections were then freed of skin, bone and turbinate, retaining only the nasal septum. The septa were washed tree times with Dulbecco's modified Eagle medium (MEM) high in glucose and were treated as shown in Tables 1 and 2. The septa were placed in 50 mm diameter, 20 mm high Petri dishes containing 12 mL MEM at 37°C in a humid incubator with an atmosphere of 5% CO_2_. After 2 h of incubation, the tissues were submerged in McDowell and Trump's fixative (4% commercial formaldehyde and 1% glutaraldehyde) in 0.1 M Sorenson's sodium phosphate buffer [27].

### 2.5. Tissue Processing

For morphological analysis, tissues were semithin sectioned (0.5 *μ*m thick). Briefly, the tissues were decalcified in 10% EDTA for 7 days, washed in buffer phosphate, and postfixed in 1% osmium tetroxide (OsO_4_). They were then dehydrated in ascending alcohols and included in Epon 812 (Polysciences, Warminster, PA). Three semithin sections were obtained from each sample and stained with toluidine blue.

Changes to the respiratory epithelium exposed to whole *B. bronchiseptica *and to *B. bronchiseptica *LPS were quantitatively and semiquantitatively evaluated. Changes such as the number of vacuolated cells, number of dead cells, desquamated cells (for descriptive morphological purposes the following were accepted as cell death criteria: pyknosis and karyorrhexis, desquamated cells in the lumen with such nuclear changes and cell detritus in the lumen), and polymorphonuclear neutrophils (PMN) infiltrating the epithelium were quantified over 120 epithelial cells with a 100x objective. A semiquantitative analysis of the activity of the goblet cells, detritus and mucus in the lumen was also performed. Four fields were examined for each tissue section.

### 2.6. Indirect Immunoperoxidase (IIP)

The presence and localization of bacteria on the respiratory epithelium were evaluated using Walker and Mayer's [28] IIP technique, with some modifications. The immunoadsorbed anti-*B. bronchiseptica *polyclonal antiserum described in Section 2.3 was used as a primary antibody; as a secondary conjugate, protein G (Sigma Biochemicals, St. Louis, MO, USA) labeled with peroxidase was used. Septa not exposed to bacteria and septa in which the primary antiserum was replaced with preimmune ovine sera were included as negative controls. Septa of rabbits suffering from a natural disease and from which *B. bronchiseptica *had been isolated and diagnosed by IIP and histopathology were included as positive controls. A final 1 : 50 dilution of the immunoadsorbed antiserum was used.

### 2.7. Lectin Histochemistry

To detect the LPS of *B. bronchiseptica *in the respiratory epithelium of the nasal septum a lectin histochemical technique was implemented [29]. Briefly, nasal septa were incubated with a lectin-enzyme conjugate which consisted of lectin from *Limulus polyphemus *(LPA) conjugated to the alkaline phosphatase enzyme (Alkaline Phosphatase Conjugated Limulus polyphemus Lectin Horseshoe Crab, EY Laboratories Inc., USA), which binds specifically to the sugar 2-keto-3-deoxyoctonate (KDO) in the core of LPS [30].

### 2.8. Statistical Analysis

Experimental error was controlled for as follows: random application of treatments, blind evaluation of tissue, independence with the Durbin-Watson test and normality with Shapiro-Wilk's test. A test for comparing means was used for the proposed hypotheses, which proved to be significant.

## 3. Results

### 3.1. Biological Effects of *B. bronchiseptica *LPS

Mice intraperitoneally inoculated with LPS showed signs compatible with endotoxemia after 36 h, including depression, nasal and ocular secretion, bristled hair, and watery feces. Macroscopically, the lungs were congested and had petechial hemorrhages in their serous membranes. Histopathologically, microcirculatory changes in the lung consisting of edema, congestion, alveolar hemorrhages and infiltration of PMN in the alveolar septa and the alveolar space were observed. In the liver, mild perivascular mononuclear inflammatory infiltrate and moderate congestion were the main findings.

### 3.2. *In Vitro *Exposure of Nasal Septa to *B. bronchiseptica*

**Figure 1:**
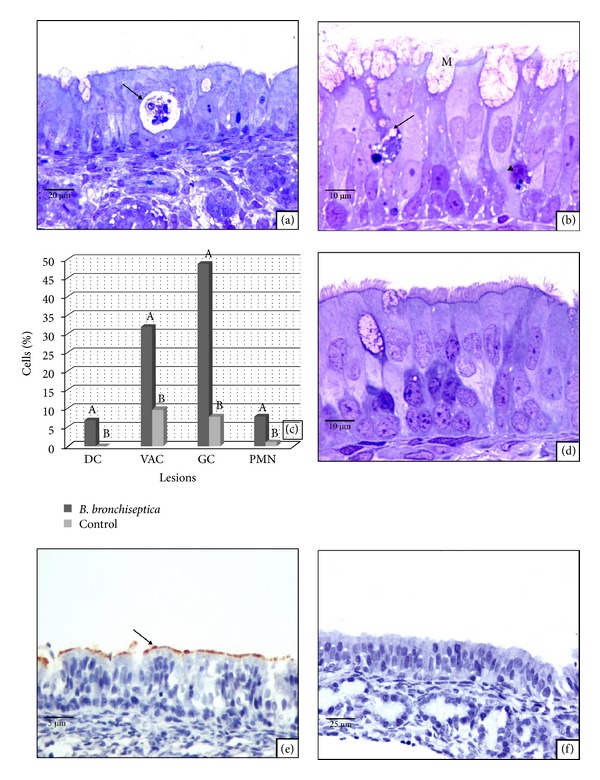
(a) Tissue culture exposed to *B. bronchiseptica *for 4 hours. Dead epithelial cells are indicated (arrow); in some cases cell death affected several cells forming intraepithelial cysts. A 40x magnified semithin section is shown. (b) Fetal rabbit nasal septum respiratory epithelium exposed to *B. bronchiseptica* for 4 hours. Increased goblet cell activity; besides the increased number of these cells, their location at different heights within the epithelium is obvious and some GC have partially protruded over the ciliated cells and released their content (M). Cytoplasmic vacuolation of other epithelial cells (arrows) and a dead cell are indicated (arrow head). A 100x magnified semithin section is shown. (c) The effect of *B. bronchiseptica *on nasal septum respiratory epithelium compared to tissues not exposed to the bacterium: dead cells (DC), desquamated cells (Des), vacuolated cells (Vac), goblet cell activity (GC), and infiltration of PMN (PMN) are indicated. Bars with different letters have statistical difference with significance level of 95% (*P* < 0.05). (d) Control fetal rabbit nasal septum respiratory epithelium tissue culture at 0 hours. A 100x magnified semithin section is shown. (e) Fetal rabbit nasal septum respiratory epithelium exposed to *B. bronchiseptica*. Positive staining of ciliated border is indicated (arrow). A 100x magnified IIP is shown. (f) Negative control fetal rabbit nasal septum respiratory epithelium not exposed to *B. bronchiseptica*. A 100x magnified IIP is shown.

Respiratory epithelial cells of the nasal septa experimentally incubated with *B. bronchiseptica *for 2 h or 4 h showed no statistically significant changes; in consequence, for statistical purposes the results of both experimental time points were grouped. However, the changes in the epithelium of exposed septa were significantly different than those at 0, 2, and 4 h that had not been exposed to the microorganism. Respiratory epithelial cell death (Figure 1(a)) and PMN infiltration into the respiratory epithelium of the septa exposed to *B. bronchiseptica *were significantly different (*P* < 0.005) than negative controls. Cellular degeneration, such as cytoplasmic vacuolation and reactive changes, such as increased goblet cell activity (defined by a larger cytoplasm of the GC, protrusion above the ciliated cells and the GC liberating their content into the lumen (Figure 1(b))), were also significantly different (*P* < 0.05) than the control groups (Figure 1(c)). Cytoplasmic vacuolation of respiratory epithelial cells was present in some of the control tissues at 0 hours but was minimal (Figure 1(d)).

### 3.3. Nasal Septa Exposed to *B. bronchiseptica *LPS

**Figure 2:**
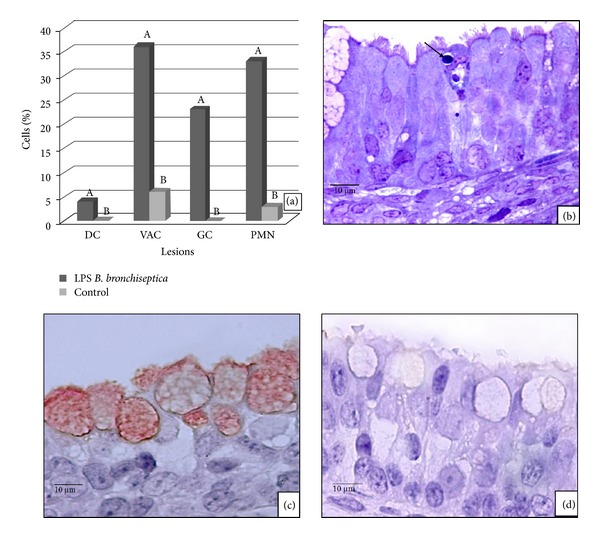
(a) The effect of *B. bronchiseptica *LPS on nasal septum respiratory epithelium compared to control explants. Dead cells (DC), vacuolation (VAC), infiltration of PMN (PMN), desquamated cells (DES), and goblet cell activity (GC) are indicated. Bars with different letters have statistical difference with significance level of 95% (*P* < 0.05). (b) Nasal septa respiratory epithelium exposed to *B. bronchiseptica *LPS. Epithelial cell death (arrow) on a 100x magnified semithin section is shown. (c) *B. bronchiseptica *LPS-treated fetal rabbit nasal septum respiratory epithelium. Positive goblet cell cytoplasm is shown by 40x magnified lectin histochemistry. (d) Negative control fetal rabbit nasal septum respiratory epithelium not exposed to *B. bronchiseptica *LPS. A 100x magnified lectin histochemistry is shown.

The main changes in *B. bronchiseptica *LPS-treated explants were cytoplasmic vacuolation of epithelial respiratory cells, infiltration of PMN into the epithelium, and increased goblet cell activity (Figure 2(a)). All of these lesions, including death of the epithelial cells, were statistically significant when compared to control tissues (*P* < 0.05) (Figure 2(b)).

### 3.4. Nasal Septa Exposed to *B. bronchiseptica: *IIP

*B. bronchiseptica *was observed attaching to the ciliated border over the course of 2 hours (Figure 1(e)). Conversely, no specific staining was found in control tissues (Figure 1(f)).

### 3.5. Nasal Septa Exposed to *B. bronchiseptica *LPS: Lectin-Histochemistry

The specific reaction of *B. bronchiseptica *LPS with lectin histochemistry was found mostly within goblet cell cytoplasm (Figure 2(c)); controls did not show a similar reaction.

### 3.6. Comparing *B. bronchiseptica *Induced-Lesions in the Nasal Respiratory Epithelium to Those Induced by Its LPS: Light Microscopy and IIP

**Figure 3:**
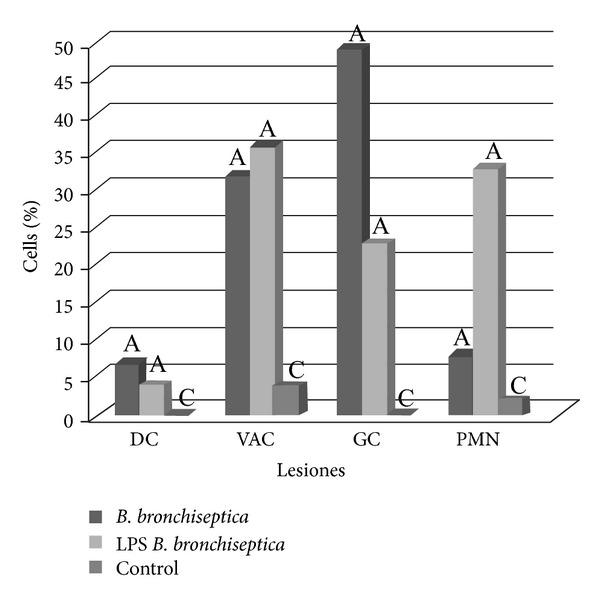
A comparison of lesions in nasal respiratory epithelium induced by *B. bronchiseptica*, its LPS, and control tissues. Dead cells (DC), goblet cell activity (GC), desquamated cells (Des), vacuolation (Vac), and infiltration of PMN (PMN) are indicated. Bars with different letters have statistical difference with significance level of 95% (*P* < 0.05).

All lesions, excluding vacuolation of epithelial cells, caused by *B. bronchiseptica *in the respiratory epithelium were more severe than those induced by its LPS alone (Figure 3). IIP immunostaining of the *B. bronchiseptica *bacteria and lectin-histochemistry of its LPS differed in their localization. However, *B. bronchiseptica *antiserum preferentially stained the ciliated border of the epithelium lectin-histochemistry of *B. bronchiseptica*, LPS stained almost exclusively within goblet cell cytoplasm (Figures 1(e) and 2(c)).

## 4. Discussion

While descriptions of the respiratory nasal epithelial lesions caused by *B. bronchiseptica *in piglets are well documented [12–14], they are less so for dogs, cats, and rabbits. Additionally, a detailed study of the cellular changes caused by some of the virulence factors of this pathogen in cell cultures has been reported [31–33]. However, to the best of our knowledge, a thorough study of the cellular changes during the initial stages of the infection and the distribution of *B. bronchiseptica *or its LPS in an isolated manner by using a model that more closely reflects the *in vivo* conditions (i.e., reconstructing the architecture and cell relationships of the respiratory epithelium in a natural host of this microorganism) has not been documented. In this work, we successfully developed a nasal septa tissue culture from fetal rabbits to study the first steps of infection with *B. bronchiseptica *and its LPS.

Tissue culture models from human trachea, lung, nasopharynx, and nasal turbinates for studying the colonization, invasion, and pathogenic effects of other microorganisms, such as *Streptococcus pneumoniae*, *Neisseria meningitides, *and *Mycobacterium tuberculosis*, have been previously developed [34–41]. Here, we cultured the nasal septum from fetal rabbits at 26-day of gestation for 4 h without major morphological changes in the epithelial cells. Importantly, we also examined the earliest changes during the interaction between *B. bronchiseptica *or its LPS with that epithelium, as that is the time during which the first steps of adhesion and colonization take place.

As early as 2 hours postexposure to *B. bronchiseptica *or its LPS, several degenerative, reactive, and deadly changes were present in the respiratory epithelial cells. These consisted of cytoplasmic vacuolation, activation of GC, infiltration of PMN and dead cells within the epithelial layer or desquamated into the lumen.

Vacuolation is considered a sign of cellular degeneration and cell death [42, 43]. A similar finding has been described *in vivo* in rabbits experimentally infected with *Pasteurella multocida *and in rabbits with natural respiratory disease [44, 45]. Nevertheless, no previous publications have documented this lesion within the respiratory epithelium of rabbits or other species during the first hours of infection induced by *B. bronchiseptica *or its LPS, using similar experimental conditions as those employed in this study.

Goblet cell activity was observed after challenge with either *B. bronchiseptica *or its LPS; the cells were enlarged due to an apparent accumulation and enlargement of their cytoplasmic content, and a higher number of GC were observed protruding above the normal apical limit of the respiratory epithelium or were found extruding their content, which led to mucus accumulation in the luminal septa. Tesfaigzi et al. [46] stated that LPS induces an increase in the number of epithelial cells in the respiratory mucosa by increasing the quantity of GC (i.e., hyperplasia). This contention has been contradicted by other authors who have proposed that the synthesis and secretion of mucus are synchronic and do not alter the number or size of GC [47, 48]. Our study was not intended to determine whether there was GC hyperplasia, metaplasia, or hypertrophy; however, the enlargement of these cells observed in the epithelia exposed to *B. bronchiseptica *or its LPS could not be explained without any increase in the size of their cytoplasm. This increase need not be due to a higher synthesis of mucin, but it is more likely due to a looser arrangement (due to hygroscopy) of the granula contents or the fusion of their membranes prior to expulsion.

The mechanism by which the LPS induces mucus production is not completely understood. However, it is known that LPS increases mRNA expression of MUC5AC, MUC5B, and IL-8 and that it stimulates the secretion of both mucins (MUC5AC up to 39%, MUC5B up to 31%) and the inflammatory cytokine IL8 [49]. *In vivo*, increased expression of genes coding for mucin and inducing mucous metaplasia due to LPS have been demonstrated [46]; the LPS stimulates inflammatory mediator synthesis by the PMN, which in turn drives the expression of the aforementioned genes. Given the short experimental time in our study, it would be quite difficult for a cell to perform some of these elaborated processes, during which the activation of several genes must take place. Typical morphological changes of cell death in human tracheal cell line cultures induced by *B. bronchiseptica *have been described [32]. Studies using *B. bronchiseptica-*infected macrophages and various epithelial cell lineages have also documented cytotoxicity in a nonapoptotic mechanism that is type III secretory system-dependent. Nogawa et al. [31] and Kuwae et al. [33] reported that the mechanism by which *B. bronchiseptica *induces necrosis is mediated by the complex BopC and BopB-BopD proteins, with tyrosine phosphatase activity translocated through the secretory type III system. The activity apparently lies in the ability of BopD and BopB to induce the formation of pores in the cytoplasmic membrane of eukaryotic cells through which other effector proteins are introduced into the cytoplasm. Morphologically, the dying cells seem necrotic rather than apoptotic, showing cytoplasmic swelling and extensive membrane blebbing without nuclear changes [50].

Qualitatively, the changes induced by the *B. bronchiseptica *LPS were similar to those caused by the bacterium. Quantitatively, however, the two differed in that, with the exception of PMN infiltration into the epithelium, the lesions were less severe with the LPS than with the bacterium. It has been previously indicated that other pathogen-associated molecular patterns (PAMPs) expressed by the bacterium, in addition to the LPS, can induce genomic expression in host cells mediated by TLRs other than TLR4. This is further supported by the observation that live *B. bronchiseptica *induces a small subset of genes that are not activated by the LPS [51, 52].

In this work, LPS induced a higher infiltration of PMN in the respiratory epithelium and the propria of nasal septa than did the bacterium. Multiple studies have demonstrated that the main inflammatory cells in tracheobronchial lavages of rats that were intranasally and intratracheally exposed to different doses (20 to 1000 *μ*g) of *Pseudomonas aeruginosa *LPS were neutrophils, with macrophages, lymphocytes, and eosinophils being absent. Such LPS doses are enough to initiate neutrophilic inflammation in the lungs [48, 53]. The dose used in our work was 10 *μ*g/mL (150 *μ*g final concentration); this dose closely resembles those reported by others [21–23]. The difference in the number of PMN induced by *B. bronchiseptica *LPS and *B. bronchiseptica *bacteria could be explained by selective activation induced by the LPS of the PMN through its PAMP recognition receptors, more specifically through TLR4. Mann et al. [51, 54, 55] and Kirimanjeswara [56] documented TLR4 activation by *B. bronchiseptica *LPS and they noted its importance for PMN recruitment and cytokine production.

Several proteins are required for the migration of a PMN from the venular lumen to its final destination in the epithelium, be it in the interstitial tissues or the respiratory track; all of these proteins were present and most likely expressed at high levels in the experimental tissue cultures employed in this study. However, the question of where the increased numbers of PMN observed infiltrating the epithelium of the septum came from still remains. This PMN infiltration cannot be easily explained in this study as the nasal septa were completely separated from the vascular bed of the fetuses. One hypothesis could be that these cells are in an inactive state within the remaining blood vessels or in the interstitial matrix of the nasal septum and that they are not recognizable until activated by the bacteria or their products. Additional studies are required to address this issue. However, the importance of PMN in LPS-mediated processes cannot be ignored.

Considering the similarity of the lesions caused by *B. bronchiseptica *and its LPS and the fact that the differences were only in quantitative terms, it is tempting to hypothesize that the lesions caused by the bacterium could simply be the result of the LPS still attached to its outer membrane. This hypothesis should be addressed in future studies. *B. bronchiseptica *and its LPS distribution on the nasal septum respiratory epithelium were evaluated by the indirect immunoperoxidase and lectin histochemistry techniques. *B. bronchiseptica *immunostaining indicated that bacteria were distributed mainly on the ciliated border of the epithelium and less frequently in detritus and mucus accumulated in the lumen. In both cases, the staining was granular in nature. On the other hand, *B. bronchiseptica *LPS was visualized mainly within GC cytoplasm. These results suggest that *B. bronchiseptica*, as well as its LPS, adhered to receptors containing glycoconjugates, be it in the glycocalyx of the interciliary space or in those produced by the GC. Filamentous hemagglutinin (FHA) of *B. bronchiseptica *is necessary and sufficient to mediate *in vitro *rat lung epithelial cell adherence [15]. The FHA of *B. pertussis *possesses a carbohydrate recognition domain (CRD) that mediates its attachment to the ciliated respiratory epithelial cells and macrophages *in vitro*. A lectin-like activity for heparin and other sulfated carbohydrates has also been identified in *B. bronchiseptica *and *B. pertussis*, which can mediate adherence to nonciliated epithelial cell lines [57–60]. Regarding the LPS, it is possible that a short duration of time is needed for it to bind to its receptors, as our experiments found LPS within GC cytoplasm in the early stages of exposure. These findings could be explained by Alexander and Rietschel's [52] results, which indicated that LBP and CD14 mediated rapid cellular internalization of high molecular weight LPS aggregates. Furthermore, Huber et al. [61] proposed that the LPS could bind soluble CD14 receptors, which would permit CD14-negative cells to respond to the LPS.

In this research, we successfully established a nasal septum culture from rabbit fetuses for studying the first steps of the relationship between *B. bronchiseptica *or its LPS with the respiratory epithelium. Models such as these, which use a complete tissue culture instead of isolated cells, more closely reflect the natural processes of an infection and allow for an improved understanding of the initial phases of the pathogen-host interaction and further examine new preventive avenues. We also demonstrated that *B. bronchiseptica *and its LPS induce very similar lesions on the septal respiratory epithelium after 2 h of exposure. The lesions in the epithelial cells were mainly degenerative, early reactive, and deadly in nature and the changes induced by the whole bacterium were even more severe. The labeling of the bacterium and its GC cytoplasm mucosubstance-associated LPS suggests that *B. bronchiseptica *and its LPS have an affinity for interciliar and GC glycoproteins, representing their respective initial adhesion locations.

## Figures and Tables

**Table 1:** Experimental protocol for fetal rabbit nasal septa exposed to *B. bronchiseptica. *

Treatment	MEM	MEM	MEM + *B. bronchiseptica *	MEM	MEM + *B. bronchiseptica *
No. of explants	3	6	9	6	9
Dose	Negative control	Negative control	10^7^ CFU*	Negative control	10^7^ CFU*
Exposure time	0 h	2 h	2 h	4 h	4 h

*[6–9].

**Table 2:** Experimental protocol for fetal rabbit nasal septa exposed to* B. bronchiseptica* LPS.

Treatment	MEM	MEM	MEM + LPS *B. bronchiseptica*	MEM	MEM + LPS *B. bronchiseptica*
No. of explants	3	6	9	6	9
Dose	Negative control	Negative control	10 *µ*g/mL*	Negative control	10 *μ*g/mL*
Exposure time	0 h	2 h	2 h	4 h	4 h

*[21–23].
